# Supplementary material for: Complex‐centric proteome profiling by SEC‐SWATH‐MS
Source: Mol Syst Biol. 2019 Jan 14;15(1):e8438. doi: 10.15252/msb.20188438 (PMC6346213; doi:10.15252/msb.20188438)
Supplement: Supplementary file 8 — Dataset EV7 [file MSB-15-e8438-s008.zip › feature_plots_string/P14324.pdf]

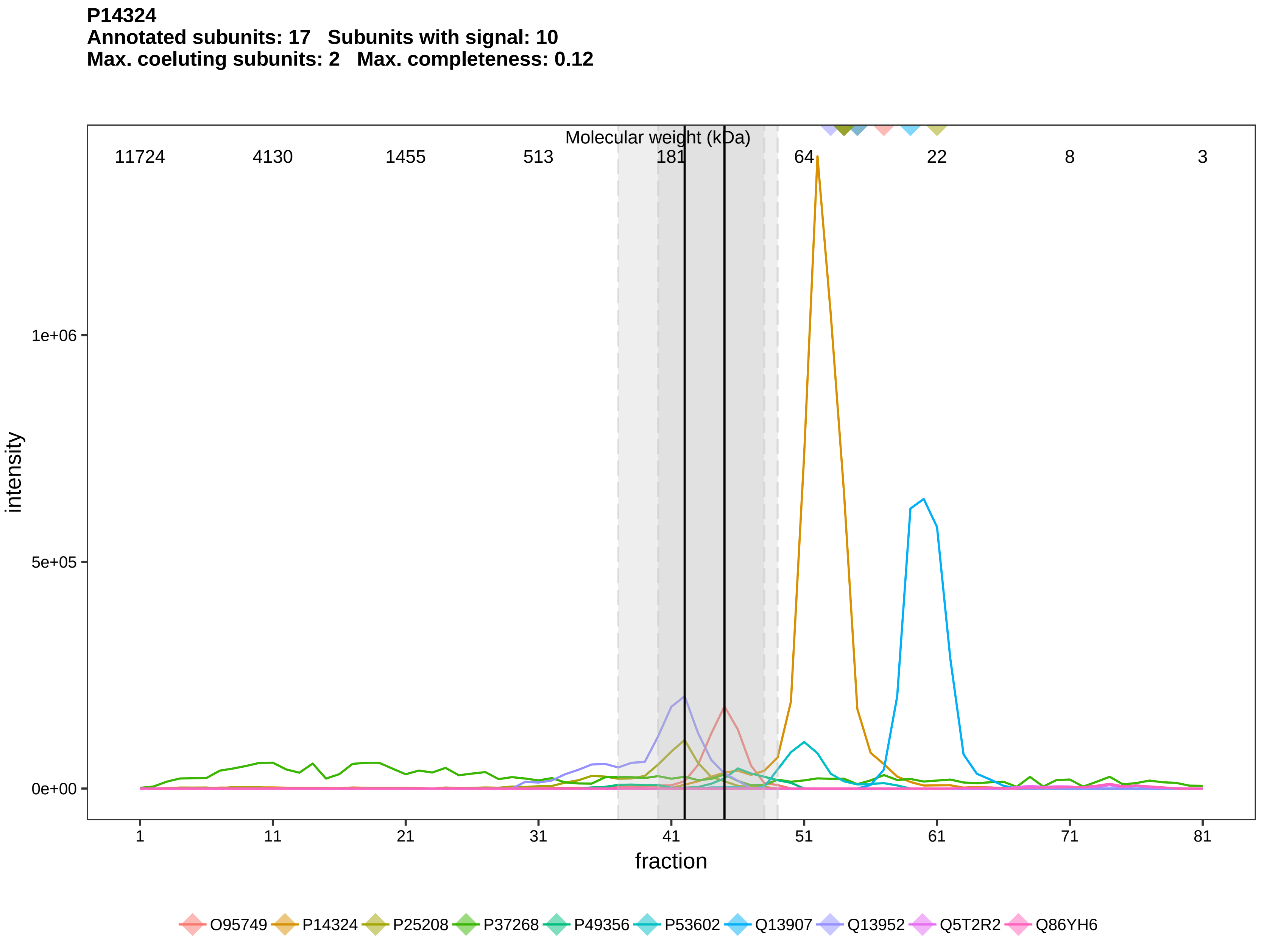
P14324
Annotated subunits: 17 Subunits with signal: 10
Max. coeluting subunits: 2 Max. completeness: 0.12
Molecular weight (kDa)
11724
4130
1455
513
181
64
22
8
3
intensity
1e+06
5e+05
0e+00
1
11
21
31
41
51
61
71
81
fraction
O95749
P14324
P25208
P37268
P49356
P53602
Q13907
Q13952
Q5T2R2
Q86YH6
